# Supplementary material for: Population modeling of tumor growth curves and the reduced Gompertz model improve prediction of the age of experimental tumors
Source: PLoS Comput Biol. 2020 Feb 25;16(2):e1007178. doi: 10.1371/journal.pcbi.1007178 (PMC7059968; doi:10.1371/journal.pcbi.1007178)
Supplement: S1 Table — Models ranked in ascending order of AIC (Akaike information criterion). Other statistical indices are the log-likelihood estimate (-2LL) and the Bayesian information criterion (BIC). (PDF) [file pcbi.1007178.s001.pdf]

**Model**	**-2LL**	**AIC**	**BIC**
Gompertz	2232	2246	2253
Reduced Gompertz	2256	2266	2271
Logistic	2315	2327	2333
Exponential	2644	2652	2656
