# Supplementary material for: Population modeling of tumor growth curves and the reduced Gompertz model improve prediction of the age of experimental tumors
Source: PLoS Comput Biol. 2020 Feb 25;16(2):e1007178. doi: 10.1371/journal.pcbi.1007178 (PMC7059968; doi:10.1371/journal.pcbi.1007178)
Supplement: S2 Table — Models ranked in ascending order of AIC (Akaike information criterion). Other statistical indices are the log-likelihood estimate (-2LL) and the Bayesian information criterion (BIC). (PDF) [file pcbi.1007178.s002.pdf]

**Model**	**-2LL**	**AIC**	**BIC**
Reduced Gompertz	2953	2961	2962
Gompertz	2953	2965	2965
Logistic	3010	3020	3020
Exponential	3097	3103	3104
