# Supplementary material for: Population modeling of tumor growth curves and the reduced Gompertz model improve prediction of the age of experimental tumors
Source: PLoS Comput Biol. 2020 Feb 25;16(2):e1007178. doi: 10.1371/journal.pcbi.1007178 (PMC7059968; doi:10.1371/journal.pcbi.1007178)
Supplement: S3 Table — Fixed effects (typical values) of the parameters of the different models. ω is the standard deviation of the random effects. σ is vector of the residual error model parameters. Last column shows the relative standard errors (R.S.E.) of the estimates. (PDF) [file pcbi.1007178.s003.pdf]

**Model**	**Parameter**	**Unit**	**Fixed effects**	$\boldsymbol{\omega}$	**R.S.E. (%)**
Gompertz	α	day^{-1}	0.718	0.166	3.87
	β	day^{-1}	0.0742	0.239	5.82
	$\boldsymbol{\sigma}$	-	[28.63, 0.078]	-	[13.71, 14.09]
Reduced Gompertz	β	day^{-1}	0.077	0.121	2.79
	k	-	9.42	-	0.293
	$\boldsymbol{\sigma}$	-	[27.32, 0.11]	-	[13.75, 10.79]
Logistic	ρ	day^{-1}	0.476	0.123	2.84
	K	mm^3	1.65e+03	0.0895	4.04
	$\boldsymbol{\sigma}$	-	[39.28, 0.11]	-	[12.63, 13.08]
Exponential	α	day^{-1}	0.403	0.111	2.73
	$\boldsymbol{\sigma}$	-	[93.69, 0.35]	-	[19.59, 15.68]
