# Supplementary material for: Population modeling of tumor growth curves and the reduced Gompertz model improve prediction of the age of experimental tumors
Source: PLoS Comput Biol. 2020 Feb 25;16(2):e1007178. doi: 10.1371/journal.pcbi.1007178 (PMC7059968; doi:10.1371/journal.pcbi.1007178)
Supplement: S4 Table — Fixed effects (typical values) of the parameters of the different models. ω is the standard deviation of the random effects. σ is vector of the residual error model parameters. Last column shows the relative standard errors (R.S.E.) of the estimates. (PDF) [file pcbi.1007178.s004.pdf]

**Model**	**Parameter**	**Unit**	**Fixed effects**	$\boldsymbol{\omega}$	**R.S.E. (%)**
Reduced Gompertz	β	day^{-1}	0.077	0.072	4.11
	k	-	9.85	-	0.919
	$\boldsymbol{\sigma}$	-	[0,0.325]	-	[0,10.2]
Gompertz	α	day^{-1}	0.758	0.087	4.15
	β	day^{-1}	0.0769	0.094	5.1
	$\boldsymbol{\sigma}$	-	[0,0.325]	-	[0,10.5]
Logistic	ρ	day^{-1}	0.404	0.047	2.36
	K	mm^3	1.18e+10	0.092	8.73
	$\boldsymbol{\sigma}$	-	[0,0.495]	-	[0,13.8]
Exponential	α	day^{-1}	0.078	0.028	6.18
	$\boldsymbol{\sigma}$	-	[0,589]	-	[0,17]
