# Supplementary figures and images for: Population modeling of tumor growth curves and the reduced Gompertz model improve prediction of the age of experimental tumors
Source: PLoS Comput Biol. 2020 Feb 25;16(2):e1007178. doi: 10.1371/journal.pcbi.1007178 (PMC7059968; doi:10.1371/journal.pcbi.1007178)

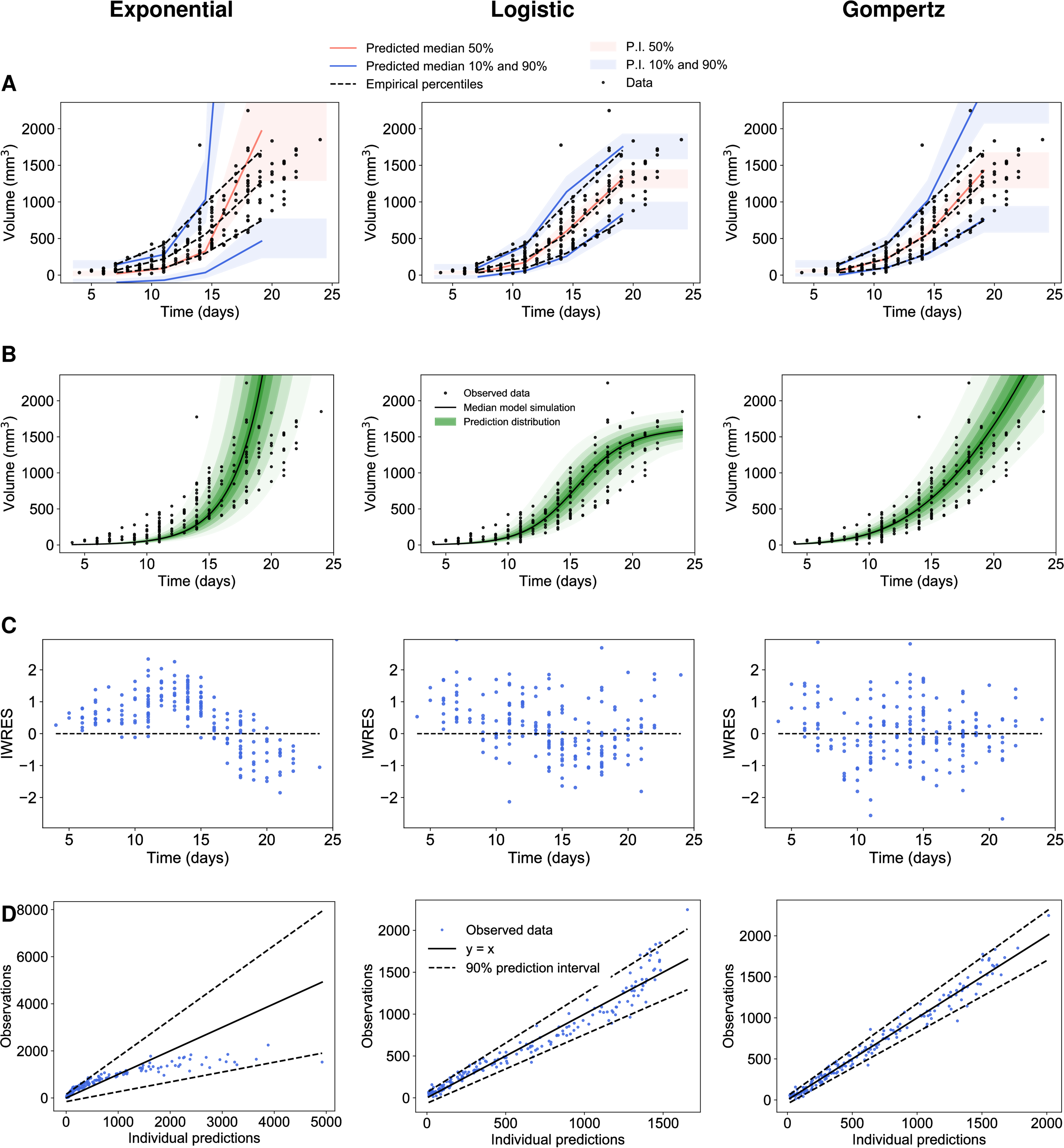

Supplement: S1 Fig — Population analysis of experimental tumor growth kinetics. A) Visual predictive checks assess goodness-of-fit for both structural dynamics and inter-animal variability by reporting model-predicted percentiles (together with confidence prediction intervals (P.I) in comparison to empirical ones. B) Prediction distributions. C) Individual weighted residuals (IWRES) with respect to time. D) Observations vs predictions Left: exponential, Center: logistic, Right: Gompertz models. (TIF) [file pcbi.1007178.s005.tif]

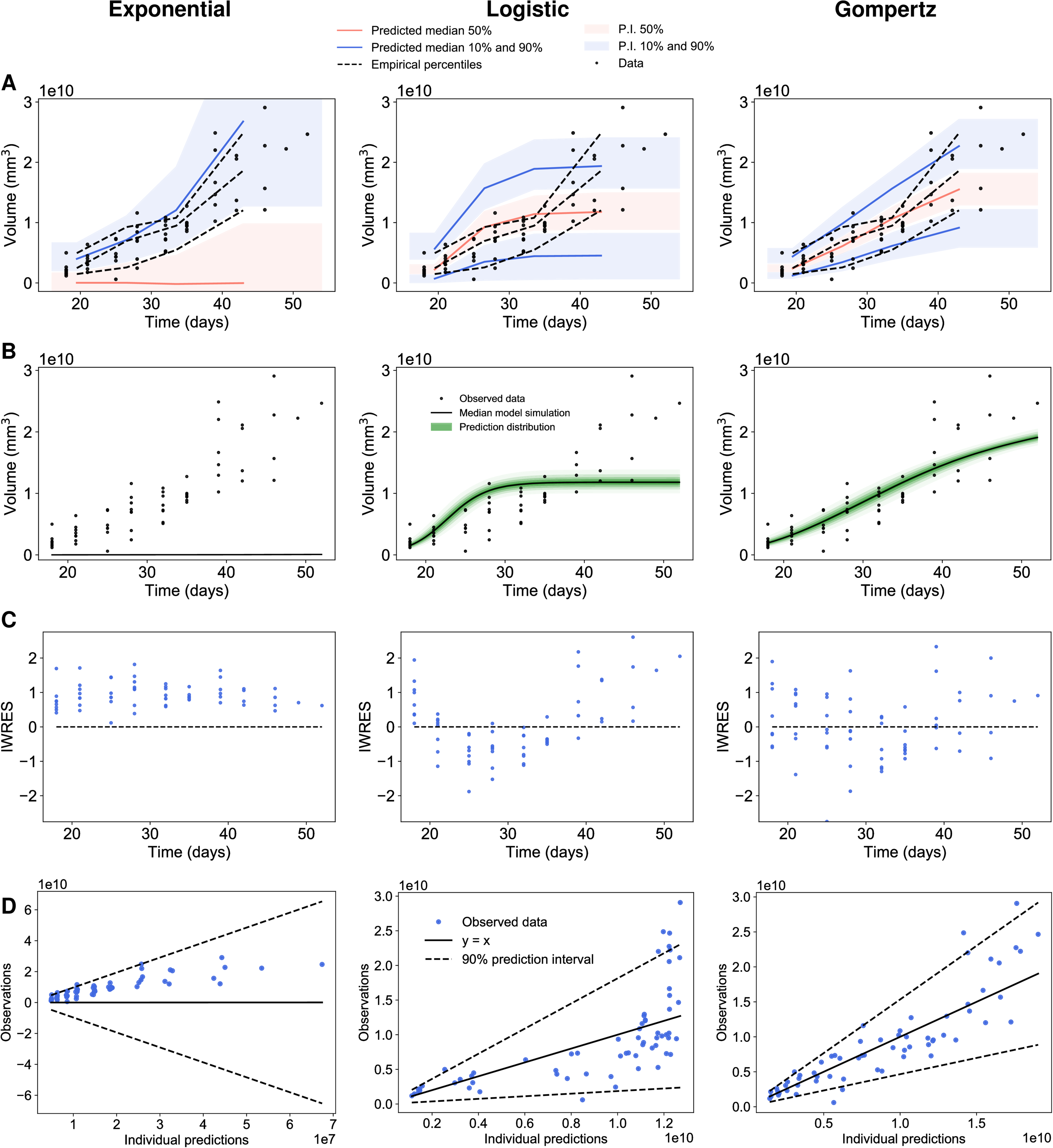

Supplement: S2 Fig — Population analysis of experimental tumor growth kinetics. A) Visual predictive checks assess goodness-of-fit for both structural dynamics and inter-animal variability by reporting model-predicted percentiles (together with confidence prediction intervals (P.I) in comparison to empirical ones. B) Prediction distributions. C) Individual weighted residuals (IWRES) with respect to time. D) Observations vs predictions Left: exponential, Center: logistic, Right: Gompertz models. (TIF) [file pcbi.1007178.s006.tif]

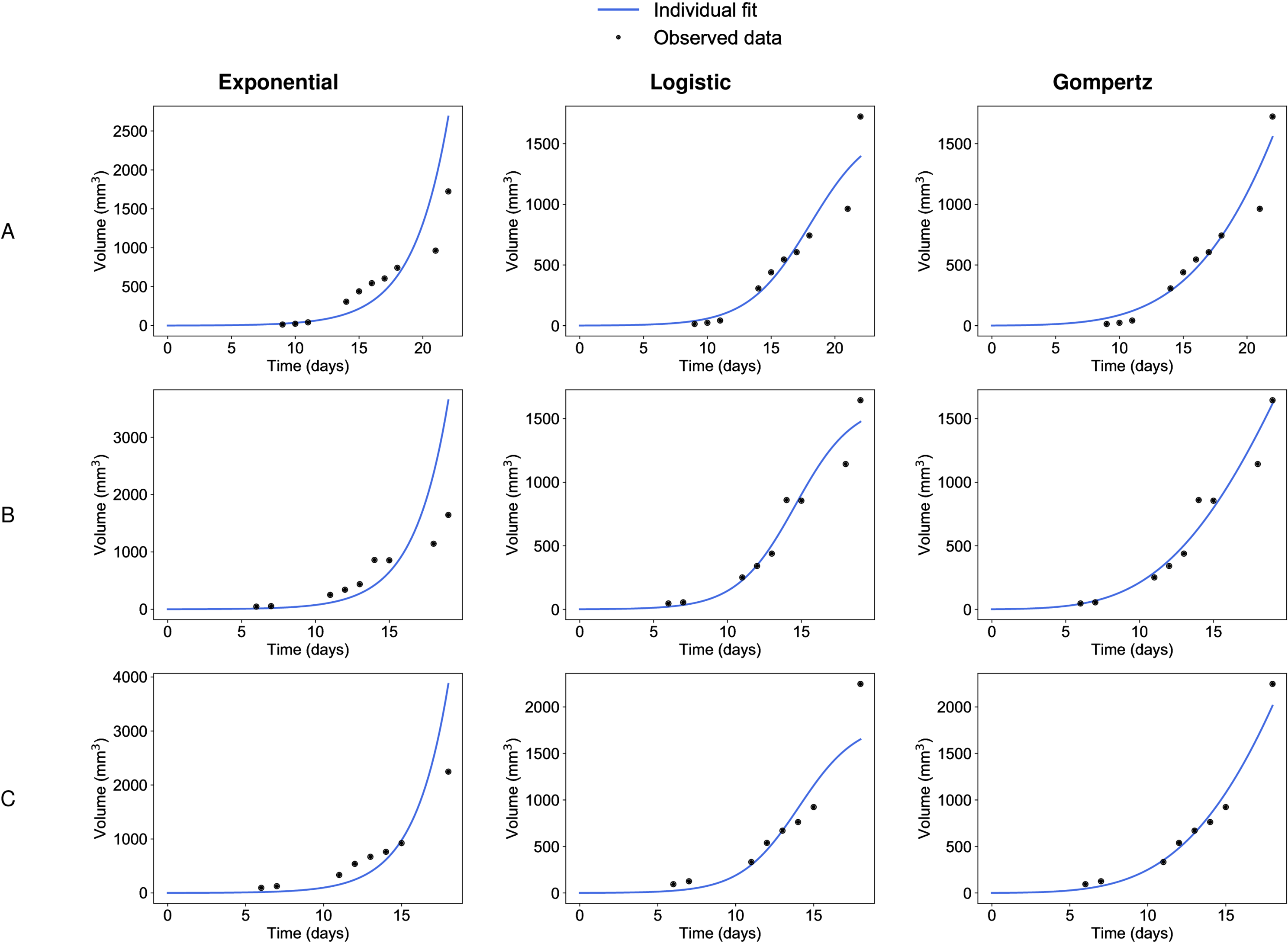

Supplement: S3 Fig — Three representative examples of individual fits (animal A, animal B and animal C) computed with the population approach relative to the exponential (left), the logistic (center) and the Gompertz (right) models. (TIF) [file pcbi.1007178.s007.tif]

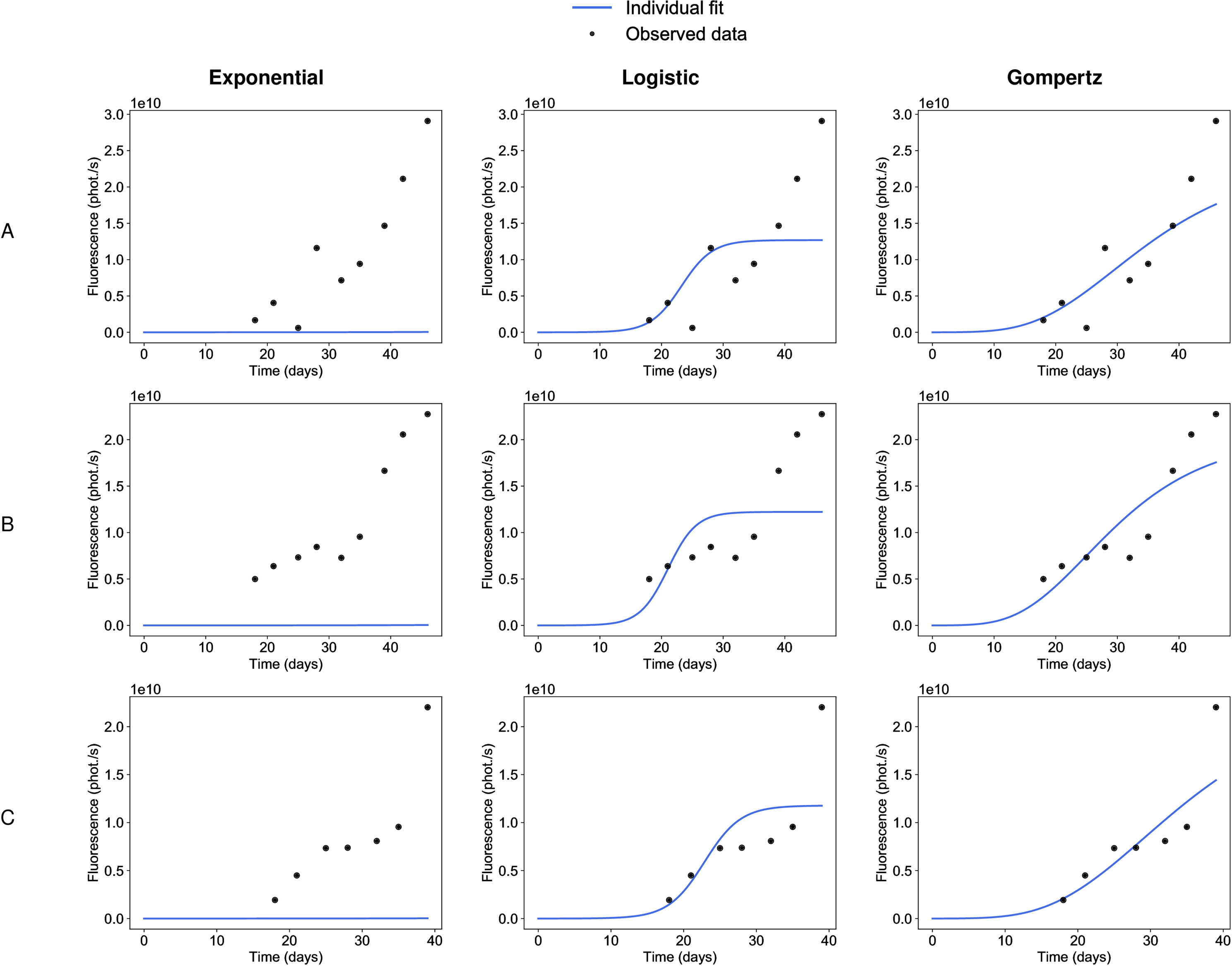

Supplement: S4 Fig — Three representative examples of individual fits (animal A, animal B and animal C) computed with the population approach relative to the exponential (left), the logistic (center) and the Gompertz (right) models. (TIF) [file pcbi.1007178.s008.tif]

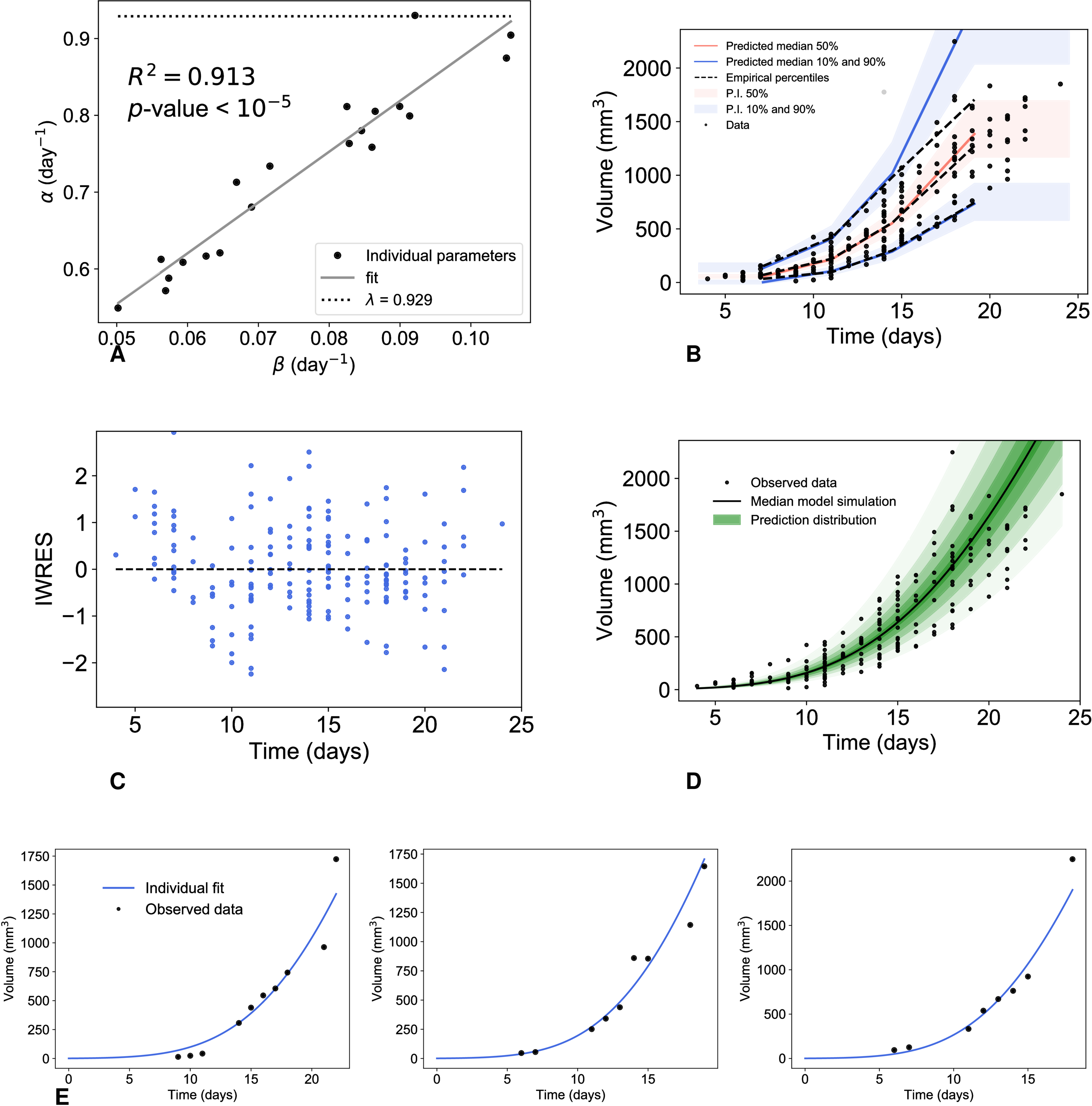

Supplement: S5 Fig — Correlation between the individual parameters of the Gompertz model (A) and results of the population analysis of the reduced Gompertz model: visual predictive check (B), scatter plots of the residuals (C), prediction distribution (D) and examples of individual fits (E). (TIF) [file pcbi.1007178.s009.tif]

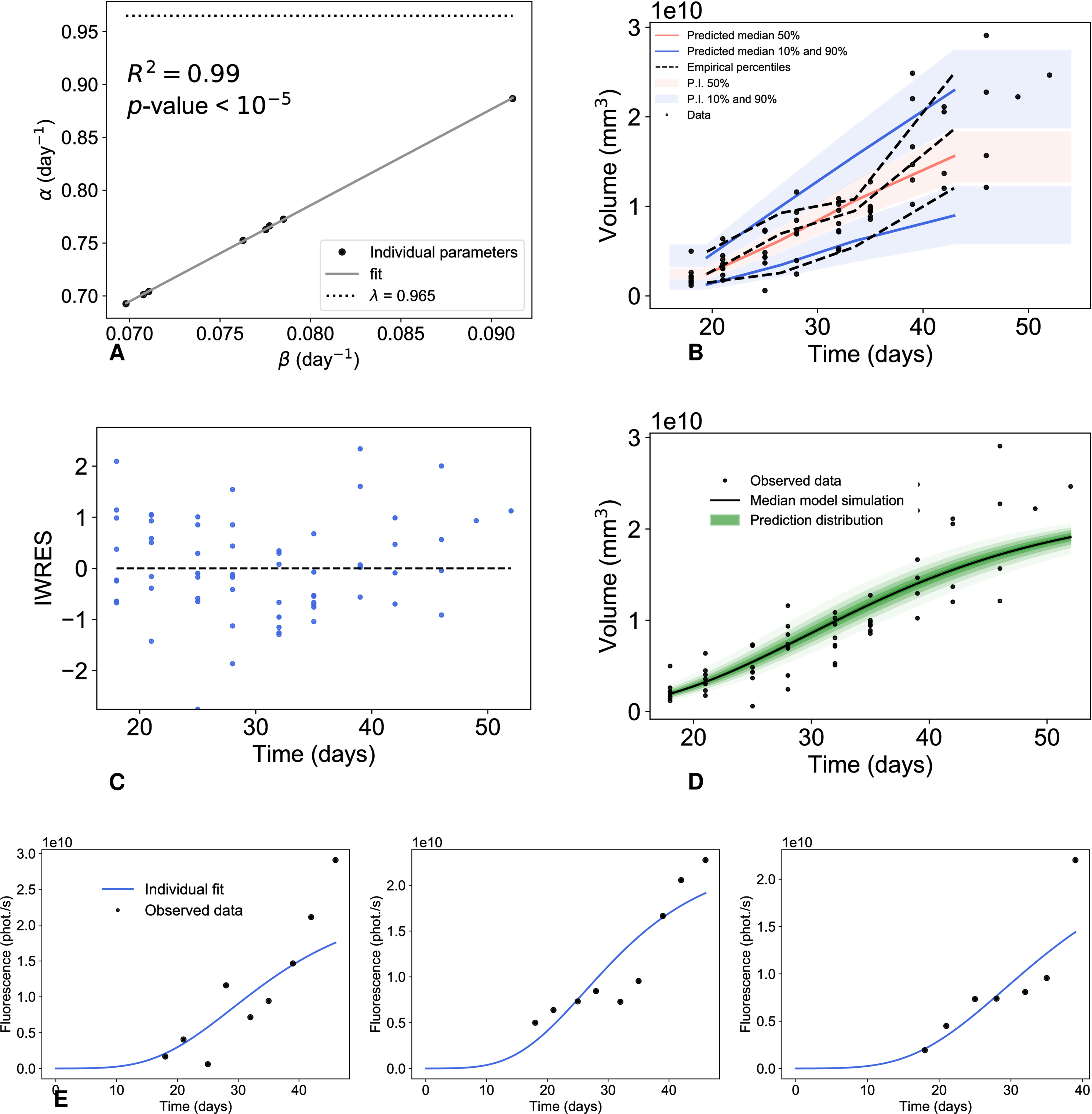

Supplement: S6 Fig — Correlation between the individual parameters of the Gompertz model (A) and results of the population analysis of the reduced Gompertz model: visual predictive check (B), scatter plots of the residuals (C), prediction distribution (D) and examples of individual fits (E). (TIF) [file pcbi.1007178.s010.tif]

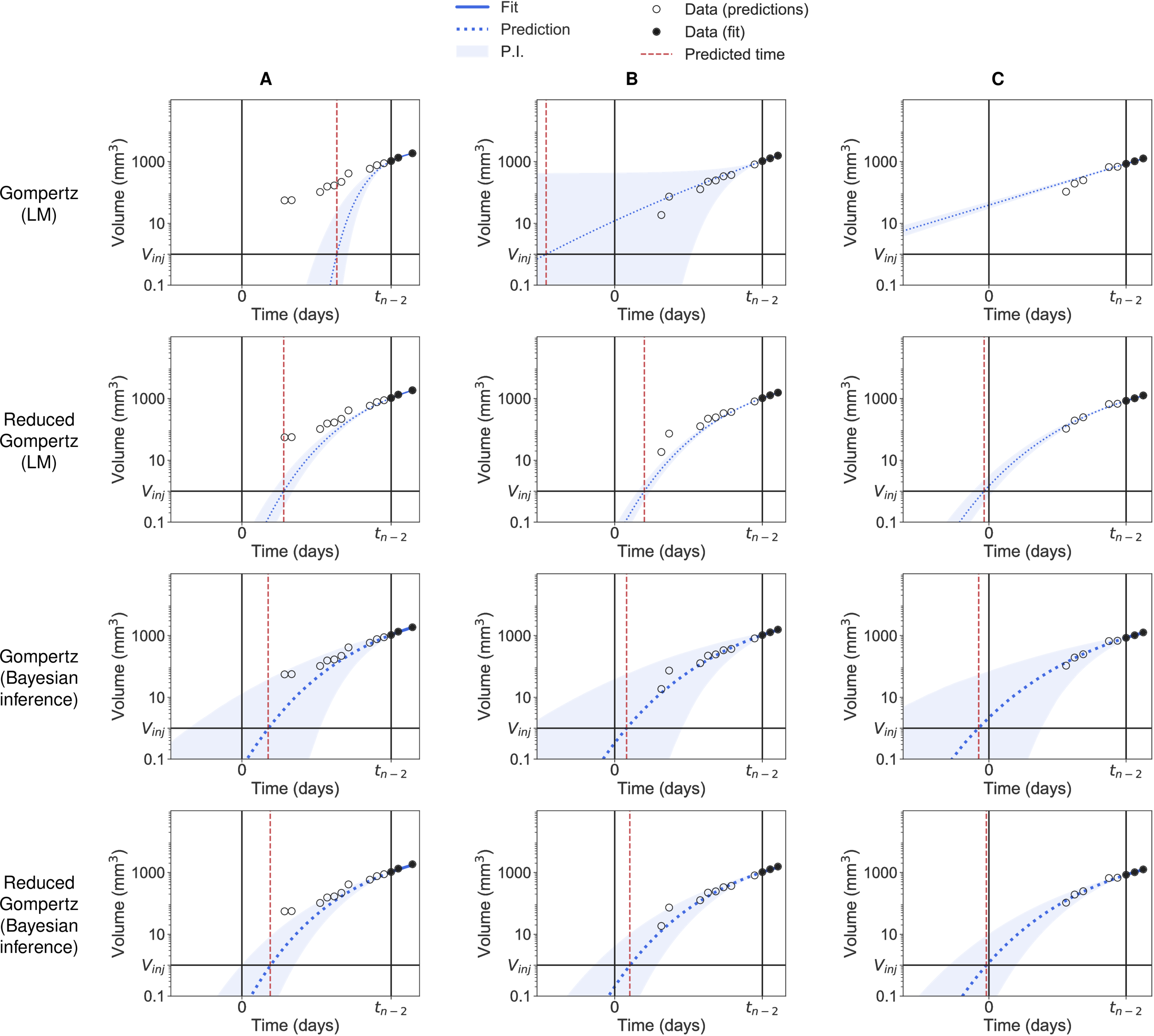

Supplement: S7 Fig — Three examples of backward predictions of individuals A, B and C computed with likelihood maximization (LM) and Bayesian inference: Gompertz model with likelihood maximization (first row); reduced Gompertz with likelihood maximization (second row); Gompertz with Bayesian inference (third row) and reduced Gompertz with Bayesian inference (fourth row). Only the last three points are considered to estimate the parameters. The grey area is the 95% prediction interval (P.I) and the dotted blue line is the median of the posterior predictive distribution. The red line is the predicted initiation time and the black vertical line the actual initiation time. (TIF) [file pcbi.1007178.s011.tif]

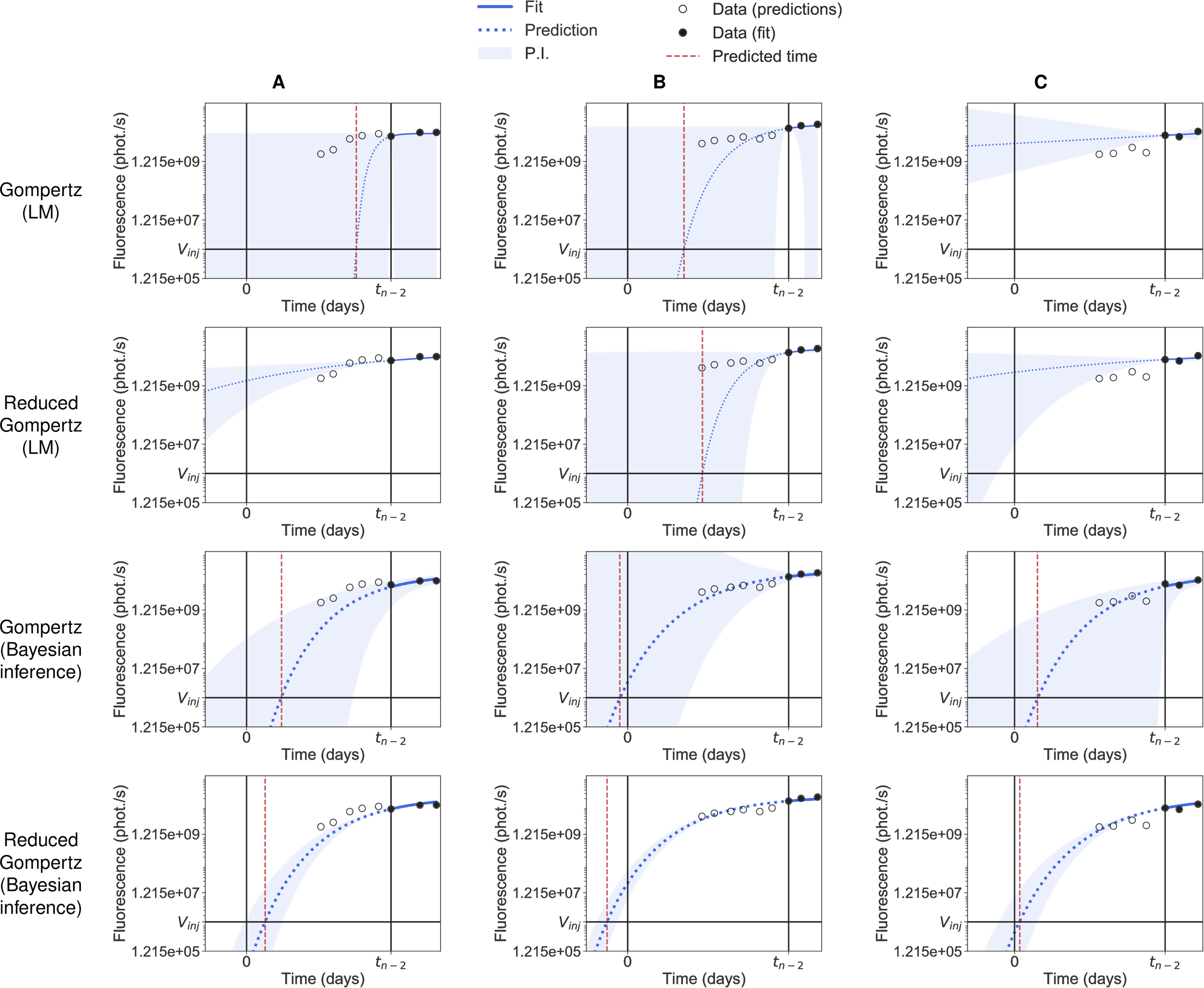

Supplement: S9 Fig — Three examples of backward predictions of individuals A, B and C computed with likelihood maximization (LM) and Bayesian inference: Gompertz model with likelihood maximization (first row); reduced Gompertz with likelihood maximization (second row); Gompertz with Bayesian inference (third row) and reduced Gompertz with Bayesian inference (fourth row). Only the last three points are considered to estimate the parameters. The grey area is the 95% prediction interval (P.I) and the dotted blue line is the median of the posterior predictive distribution. The red line is the predicted initiation time and the black vertical line the actual initiation time. (TIF) [file pcbi.1007178.s013.tif]
