# Supplementary material for: Population modeling of tumor growth curves and the reduced Gompertz model improve prediction of the age of experimental tumors
Source: PLoS Comput Biol. 2020 Feb 25;16(2):e1007178. doi: 10.1371/journal.pcbi.1007178 (PMC7059968; doi:10.1371/journal.pcbi.1007178)
Supplement: Attachment — Submitted filename: Revision PCOMPBIOL-D-19-00943.docx [file pcbi.1007178.s015.docx]

The present manuscript reports a study of tumour growth using classical ODE models and in vivo data, in the mixed-effects modelling setup.

The authors compare three model types (exponential, logistic and Gompertz), perform population model fit and model selection, using standard goodness-of-fit criteria and visual comparison with data. Gompertzian model is found to provide best fit, and further it is suggested that one of the parameters (related to carrying capacity) can be fixed in the population, leaving only one parameter (related to the growth rate change) to have inter-individual variability, the model termed “reduced”.

The model is applied to evaluate the tumour initiation time from the last three measurement points of an animal: namely, these three points are used to evaluate individual parameters and the tumour size is back-traced to the known initial volume in order to evaluate the time. The best results are achieved using the reduced model with population priors and bayesian estimation of the individual parameters.

Eventually, the authors claim that this methodology can be used to evaluate “age of tumour”, i.e., time from its initiation in the clinical settings (while acknowledging that direct verification of this estimation is impossible).

This work is an interesting and well-designed exercise in data-driven modelling and model selection. The authors apply sophisticated methodology and derive various insights and observations. Yet, I have reservations on some of the suggested interpretations, and think that part of the conclusions and claimed prospects are too far-fetched.

It indeed can be interesting to evaluate the utility of different growth models for describing data in different contexts. However, I would not take the advantage of Gompertz model in this study to signify its universal prevalence, even for the in vivo data only. First, a proper comparison could include more flexible alternatives; e.g., both logistic and Gompertz are special cases of a generalized model (with an additional parameter). It is conceivable that such general model could provide better fit to data, in tebs of AIC/BIC, notwithstanding additional degrees of freedom. Another possibility would be to evaluate the general model and to check whether the evaluated parameters indeed suggest that it is closer to the Gompertz formulation, rather than to pure logistic.

Second, The authors themselves suggest the possible properties of the data that make them more suitable for Gompertz curve – the way the exponential velocity decelerates makes the symmetric logistic curve less suitable. However, from the presented individual plots, it seems that the overall pattern is that the logistic model reaches early saturation, while Gompertz is almost not saturating at all. This suggests two questions: (1) did the authors verify that the parameter ranges for the logistic model allowed obtaining such late saturation (higher K and larger alpha)? (2) could it be that the model with decelerating growth but no saturation (e.g., V~t^alpha for alpha ~1-2) would provide also a good description of these data? Inspection of the pooled data charts suggests that the latter is a viable option.

Further, it is known that there is a certain delay time between the xenograft implantation and initiation of its growth, just due to the time required for the cells to adjust to the new environment (and possible death of part of these cells). This is not taken into account here explicitly, and hence the Gompertz model gets its advantage from the possibility to accommodate this lag, which is not formally included between its assumptions.

Considering the so-called “reduced Gompertz model” – I would not call it a reduced model, in order to not give a false impression that this is model with less parameters or different structure, while the whole change amounts to reducing the random component in the population model for one of the parameters. Further, if one used a parameterization with beta and K, the equation becomes

dV/dt = beta*ln(K/V)*V,

from which it is clear that beta is the parameter that guides the growth rate (and not only alpha, as is claimed in the article).

It is indeed an interesting observation that the parameter K (or equivalently, k) can be fixed in the population. However, given that this happens in the set of identical mice implanted with the same amount of identical tumour cells, in one experiment, this is hardly surprising. It would be far-fetched to conclude that K is a species / tumour type constant (and indeed, as noted by authors, it differs in the three analyzed datasets).

Eventually, the translation of the ability to trace the history of tumor back from this highly uniform and controlled experimental setting (all in all, the individual tumours seem to grow very similarly and synchronously) into any clinical application is extremely uncertain. There is yet no well-settled theory on the typical individual history of human cancers, which may well differ from the case when the already significantly transformed cancer cell is implanted into an experimental animal. In simple words, the “growth law” followed by a clinical tumour can change significantly (both in parameters and structure) during disease progression, especially at early stages. Therefore, I suggest to moderate the optimistic prospects of using this approach in the clinical setup.

More technical remarks and suggestions:

1. p. 3 l. 82-85: please check formatting (superscript)

2. p.3 l.109: alpha is not equal to the specific growth rate at t=t_I or t=0 in the logistic equation. In this paragraph it is also not clear what is the relation between t_{inj}, V_{inj} and t_I, V_I

3. p.4 l.130: the choice of the log-normal model for the parameters should be motivated

4. p. 4 l.134-137: for the reader who is not intimately familiar with the NLME field, there is not enough information here; please give some more details and add a reference to a good theoretical literature source

5. p.4 l. 143-144: why do you need to shift the time scale?

6. p. 5 l.154: “constant part σ1 was found negligible” there is no sufficient information to support this claim. Please explain how you evaluated this from Table 2. I have a suspicion (and indeed you mention this in the discussion) that the goodness-of-fit comparison is influenced by your choice of error model. Using proportional error, naturally emphasizes smaller values more, comparing to the additive model. Did you try to fit the data using only additive error?

7. p. 5 l.163: please explain the formula for \hat{s}^{2,i}. is it the sufficient statistics for the error? Why the 3-p in the denominator?

8. p. 5 l. 172: it is not clear whether V_I was sampled once per animal, or at each iteration of bayesian estimation of \theta_I

9. p.8 legend to Figure 1: please add explanations to A and (especially B). how did you generate the distributions?

10. p.10 legend to Table 2: what is CV? is it estimation of \omega?

Further, since all the parameters are estimated in log scale, it’s not clear, are these CV and RSE in the log scale or the real scale (and in the latter case they would be asymmetric)

11. p.10 l. 253: “The other data sets gave analogous results” Not clear, does it also mean, the same value of k? Or the fact that it can be fixed?

12. p. 10 Section 3.2.2 “Biological interpretation…”

I have several reservations for this discussion. First, if your suggestion is assumed, the V_{inj} at the initial stage (at t=t_{inj}) should in fact be set equal to \hat{V}_{inj} and the value of alpha should be re-estimated. At least, this is what you assumption entails.

On the other hand, leaving the parameters as they are, one could hypothesize that in the new conditions the cells reduce their division rate (which also sounds more plausible, from the biological point of view). Cf. my remark above about the lag in the growth after the implantation. I think this merits more elaborate analysis.

13. p. 12 Figure 3 legend: I suspect the panel enumeration got mixed. The same is true for figures S5,S6

14. p. 14 Table 3 legend: please explain the PI column

Recommendation: I think the work is good and merits publication, after the above suggestions and concerns are addressed
