# Supplementary material for: Population modeling of tumor growth curves and the reduced Gompertz model improve prediction of the age of experimental tumors
Source: PLoS Comput Biol. 2020 Feb 25;16(2):e1007178. doi: 10.1371/journal.pcbi.1007178 (PMC7059968; doi:10.1371/journal.pcbi.1007178)
Supplement: Attachment — Submitted filename: revision.docx [file pcbi.1007178.s016.docx]

**Zvia Agur, PhD
Guest Editor
PLOS Computational Biology

Feilim Mac Gabhann
Editor-in-Chief
PLOS Computational Biology**

Dear Editors,

Thank you for your consideration of our work and efforts dedicated to its evaluation. We would also like to thank the reviewers for their valuable comments and suggestions, which helped us improve the manuscript. We have made substantial revisions and hope that it can now be considered for publication in PLOS Computational Biology.

Major changes in the revised version include:

1. We now provide the entire code and data sets that was used for the modelling analysis and Bayesian predictions as an open-source software available at:

https://gitlab.inria.fr/cvaghi/reduced_gompertz_backward_predictions

2. In the backward predictions we have considered a combined error model instead of a proportional one. In the previous version we claimed that the constant part of the error model was negligible, but this is not true when the last three measurements are small. Table 3, Figure 4 and Figure 5 have been updated with the new results. They do not differ significantly from the previous results, confirming that in our case the constant part of the error model could have been considered negligible. However, we chose to change it to generalize our methodology.
3. In the introduction we have added a review of nonlinear mixed effects modelling applied to biological and oncological problems and emphasized the novelty of our work:

*“Mathematical models for tumor growth have been previously studied and compared at the level of individual kinetics and for prediction of future tumor growth [15, 16]. However, detailed studies of statistical properties of tumor growth models using a population approach (i.e. integrating structural dynamics with inter-subject variability [17]) are rare [18]. Nonlinear mixed effects modeling of the Gompertz model has been applied to several fields in biology, e.g. to model growth in Japanese quails [19] or broiler chicken growth [20]. In the field of tumor growth modeling, studies using a population approach have mostly been conducted for perturbed tumor growth under the action of therapeutics (see e.g. [21] for a clinical study and [22] for a review). In a previous publication, our group has used a mixed-effects framework to compare the descriptive power of several unperturbed tumor growth models, yet without reporting visual predictive checks, analysis of residuals nor values of the population parameters (typical values and standard deviations of the random effects) [15]. Other related works include the coupling of tumor growth models with metastatic spreading [23, 24], or an analysis of tumor growth kinetics from different cell lines using the Simeoni model only [25, 18]. Here we go beyond these previous studies not only by providing a detailed and comparative analysis of statistical properties of multiple classical tumor growth models within a population framework, but also by extending this analysis to a larger data set (94 animals in total, including two animal models and two methods of quantification of tumor size, versus 54 animals in [15]). The main focus and novelty of the work reported here is to analyze the above-mentioned correlation between Gompertz parameters using a population approach, in order to improve model-derived predictions. This led us to a simplified model with only one subject-specific parameter (and a population-specific one), the "reduced Gompertz" model [11].”*

4. We have extended and clarified our biological interpretation of the findings in terms of the *in vitro* proliferation rate (see section 3.2.2)
5. In the discussion we have analyzed the limitations of our work:

*Our methodology might face multiple challenges for future clinical applications. First, it is indeed clearly difficult to fully characterize tumor kinetics in humans and only few studies support the evidence that it follows a gompertzian growth [8]. This is due to the limited number of available observations in the clinic and to the fact that saturation of human tumors is almost never reached, since it coincides with an advanced stage of the cancer where patients usually receive a treatment. Moreover, human tumor growth curves, even if limited to the same organ and histological type, might show a substantially larger variability than in in vivo experimental settings where cells from immortalized cancer cell lines are injected in genetically identical mice. Here, we have proven that a group of animals with similar features (such as tumor type) is characterized by a common tumor growth constant, that defines the saturation point. In the human setting, it could be interesting to analyze this constant as a function of some covariates (such as weight, sex, tumor type). Eventually, in the Gompertz model we haven’t considered that the initial phase of tumor growth might be affected by intrinsic stochasticity. Our choice was motivated by the large number of injected cells (of the order of 106) that allowed us to consider the initial variability to be negligible. For accurate clinical translation stochasticity should ideally be taken into account to model the initial stages of tumor growth.*

You’ll find below our point-by-point response to the reviewer’s comments.

Moreover, we provide a supplementary file for the reviewers including (i) an analysis of the generalized logistic model and the power law model, (ii) a statistical convergence analysis of the logistic model and (iii) of the generalized logistic model, and (iv) an analysis of the delayed Gompertz model (Gompertz model with delayed time-to-initiation).

**REVIEWER #1**

**Dear Professor Agur, the manuscript by Benzekry and coworkers is very interesting and insightful but it needs some important improvements. Thus, I suggest major revisions. The following is a list of key points to be reworked:**

**1. I liked a lot the basic idea to use mixed models as a way to relate the single individual laws of growth population-wide data. However, I disagree with the choice of comparing the Gompertz model only with the simplest two other ODE models of tumor growth: the exponential and the classical logistic model. The comparison must be done also with respect to more realistic models such as: the generalized logistic (V’ = a V – b V^{n} with 0**

We thank the reviewer for this suggestion. The main reason why we did not include the generalized logistic model is that our previous work [Benzekry et al., PLoS Comp Biol 2014] had shown that already on individual tumor growth curves this model: 1) suffered from lack of practical identifiability of the parameters (large standard errors of the estimates) and 2) exhibited very small values of the power parameter, denoted *n* by the reviewer, thus suggesting convergence to the more parsimonious Gompertz model.

Nevertheless, the reviewer’s comment motivated us to reassess these results in the context of a population approach. We thus provide a detailed analysis of the generalized logistic model in a supplementary file. We found that, although able to satisfactorily describe the data (Table R1 and Figure R2, supplementary material for reviewers), the generalized logistic model, with one parameter more than the Gompertz and logistic models, lacked robustness of convergence. Indeed, starting from different initial guesses of the parameters, the SAEM algorithm converged to different estimates. This indicates non-identifiability of the parameters. Moreover, as can be observed in Table R2 and Figure R3, the inferred value of the generalized logistic power (denoted $\nu$) is very small, suggesting that the generalized model degenerates to a Gompertz expression. Indeed, when $\nu\to0$, the generalized logistic model converges to the Gompertz model.

Finally, the main focus of our work is rather the analysis of the Gompertz model and its simplification, the reduced Gompertz model, in order to improve the robustness of parameter estimation and predictive power. This is the reason why we compared it only to the exponential and the logistic models, these being considered widely used standard growth models.

In the discussion we have added the reason why we haven’t considered the generalized logistic model in our analysis:

*“The logistic and Gompertz models belong to the same family of tumor growth equations*

*and can be seen as specific cases of the generalized logistic model* $\frac{dV}{dt}=\alpha V\left( 1-\left( \frac{V}{K} \right)^{\nu} \right)$ *[40, 15]. We also analyzed the latter model, which demonstrated good descriptive power but lacked robustness of convergence (data not shown). Indeed, the SAEM algorithm converged to different estimates starting from different initial guesses of the parameters. This might be explained by the larger number of parameters (3) that led to identifiability problems. In addition, we found that values of ν able to describe the data were often very small (< 10−3), thus suggesting convergence to the Gompertz model.”*

**2. The fact that the classical logistic law V’= p V – q V^2 did not perform well is not so surprisingly. See for example some consideration at the end of the section 2 of d’Onofrio, Chaos, solitons and Fractals (2009): the classical logistic model might correspond to space-independent cellular interactions, which are non-physical.**

We thank the reviewer for the comment and for pointing this reference, which has been added to the discussion:

*Indeed, the logistic model relies on space-independent cellular interactions, which might be biologically unrealistic [42].*

**3. The authors have detected a correlation between the parameters alpha and beta. If I well understood what the author mean, this correlation is not new but has been reported in literature in the seventies. See, for example, section 5.5 of the Wheldoin’s book (ref 40). This is not explicitly said in the manuscript: the authors simply cite references 11 and 26 and only in the discussion they list a series of previous similar results. The fact that this correlation is something previously known in literature must be explicitly written in the revised version where the correlation is described, not only in the discussion.**

The reviewer is correct that this correlation is not a new finding. We thought having been explicit about this point by already stating this fact at the second sentence of the abstract (“*In analyzing the Gompertz model several studies have reported a striking correlation between the two parameters of the model*”), as well as dedicating the second paragraph of the introduction to reference of these previous studies (lines 49-55).

Nevertheless, we have added some references when we introduce the correlation between the parameters in Section 3.2.1.

**4. Are the authors sure that the reduced Gompertz model has not been defined previously?**

The reduced Gompertz model arises from the application of the population approach to estimate the parameters, where one parameter has only fixed effects within the population and the other one has random effects. In other works [10,11] the same relation between the parameters has been included, but only individual fits have been performed. Nevertheless, we clarified the novelty of our work in the introduction:

*“The main focus and novelty of the work reported here is to analyze the above-mentioned correlation between Gompertz parameters using a population approach, in order to study the implications in terms of improvement of predictions. This leads us to use a simplified model with only one subject-specific parameter (and a population-specific one), the "reduced Gompertz" model [11].”*

We also now explicitly stipulate in the discussion that the reduced Gompertz had been stated previously in [11], and explain how our results go beyond:

*“This model has been proposed before in the context of individual tumor growth curves [11, 39] but here we leveraged the population approach to ensure reliable estimation of the population-level parameter and statistical distribution of the individual-level parameter. Importantly, while previous studies had only investigated the resulting predictive power in only one animal [10] or using simulation data [39], here we rigorously demonstrated how the reduced Gompertz allows better backward (or forward, although not reported here) prediction of tumor size and time of initiation. This analysis was performed using state-of-the art techniques from predictive modeling (e.g. cross-validation), on a large number of animals.”*

**5. In non-oncological literature on biological growth there is a number of works that apply nonlinear mixed effects modeling to Gompertz model. They ought to be mentioned and reviewed in the introduction of the revised version of the manuscript.**

We have added in the introduction a review of some works including nonlinear mixed effects modelling to the Gompertz model (see point 7).

**6. There is also some works applying mixed-effects statistical modeling to investigate tumor growth described by simple ODEs models, including the Gompertz and other ode-based models (e.g the above mentioned paper by Ribba et al or the paper by Hartung et al cancer research (2014) and, of course, Benzekry et al, 2014). Indeed, currently the authors only generically wrote “However, to our knowledge, a detailed study of statistical properties of classical growth models at the level of the population (i.e. integrating structural dynamics with inter-animal variability) yet remains to be reported. Longitudinal data analysis with nonlinear mixed-effects is an ideal tool to perform such a task [17, 18].” Thus, the authors ought to clarify in detail which are the differences between their study and the previous ones (not limited to the above-mentioned papers….)**

In the introduction we have added more details on the differences between our study and previous ones (see point 7).

**7. Summarizing points 3, 5 and 6: my feeling is that a substantial effort is needed to compare the present work to previous literature, explicitly stressing what is really new in this work (a lot) and what it has been previously published.**

This work can be seen as a continuation of the analysis introduced in [15] where we used the a priori information provided by the population approach to perform forward predictions.

Here, we report a population analysis including parameter estimation and details of the performances of the different models (in terms of diagnostic plots and identifiability metrics at the population level). Furthermore, the main novelty of the current study is to exploit the population approach to introduce a “reduced Gompertz” model with only one individual specific parameter. This allows to improve the predictive performances of the Gompertz model.

We have added in the introduction a more detailed presentation of the previous literature and novelty of this work (paragraph 3, see point 3 of our general answer to the Editor).

**8. In my opinion, the description of the Bayesian inferences ought to be more detailed. This would help both those that are more oriented to the frequentist approach in Statistics, and those (e.g. biomathematicians) who are not expert at all in statistics**

We have added some details on the Bayesian approach that we have considered. We also now provide freely the documented source code used for the analysis, which should help interested readers to reproduce the results and perform Bayesian inference. We also suggest reference [27] for further details. **9. As far as the “tumor age” is concerned, I would be much more prudent due to the fact that tumour growth is both affected by intrinsic stochasticity (of course, especially in the initial stages of growth) and also extrinsic stochasticity. What the authors brilliantly computed is merely an estimation of the average tumor age, and the associated confidence-credibility intervals of that estimate. This is something radically different from inferring the probability density of the random variable “tumor age”, which can only be done in the framework of a stochastic tumour growth model**

We thank the reviewer for this interesting observation. Indeed, intrinsic stochasticity has not been taken into account in our analysis since the number of injected cells is 10^6^, therefore we considered the initial variability to be negligible. However, this could represent a limitation in clinical applications. Extrinsic stochasticity was included in the structural error, that takes into account parameter uncertainty and the measurement error.

We have added in the discussion that intrinsic stochasticity might represent a limitation of our study (see point 10).

**10. In the discussion the authors must add a detailed discussion of the limitations of their work.**

We have added in the discussion the limitations of our work, including in particular translation to clinical applications:

*“Our methodology might face multiple challenges for future clinical applications. First, it is indeed clearly difficult to fully characterize tumor kinetics in humans and only few studies support the evidence that it follows a gompertzian growth [8]. This is due to the limited number of available observations in the clinic and to the fact that saturation of human tumors is almost never reached, since it coincides with an advanced stage of the cancer where patients usually receive a treatment. Moreover, human tumor growth curves, even if limited to the same organ and histological type, might show a substantially larger variability than in in vivo experimental settings where cells from immortalized cancer cell lines are injected in genetically identical mice. Here, we have proven that a group of animals with similar features (such as tumor type) is characterized by a common tumor growth constant, that defines the saturation point. In the human setting, it could be interesting to analyze this constant as a function of some covariates (such as weight, sex, tumor type). Eventually, in the Gompertz model we haven’t considered that the initial phase of tumor growth might be affected by intrinsic stochasticity. Our choice was motivated by the large number of injected cells (of the order of 10^6^) that allowed us to consider the initial variability to be negligible. For accurate clinical translation stochasticity should ideally be taken into account to model the initial stages of tumor growth.”*

**Kind Regards,**

**A Referee**

**REVIEWER #2**

**The manuscript considers an interesting problem of predicting tumor age. The main model used for the data analysis is the Gompertz model and its reduced form. This is correct from my point of view. The reduced version is well argued.**

We thank the reviewer for his/her positive appreciation of our approach.

**1. On the other hand, the authors compare the classic logistic model with constant carrying capacity (which should be understood as maximal tumor size here) with the form of Gompertz model in which carring capacity depends on initial data (as it is in the original Gompertz model). Typically, for the comparizon the Gompertz model is rescaled in such a way that there is a carying capacity independent of the initial value, and then the comparizon between the models makes sense. The authors should address this problem in their manuscript.**

We thank the reviewer for this interesting comment. Actually, all the parameters do not depend on initial data. Indeed, the Gompertz model reads as follows:

$\left\{ \begin{aligned} \frac{dV}{dt}= \left( \alpha-\beta\ln\left( \frac{V}{V_{inj}} \right) \right)V, \\ V\left( t_{I} \right)=V_{I} \end{aligned} \right.$	(1)

where $V_{inj}$ is the volume relative to the number of injected cells. ${(t}_{I},V_{I})$ is the initial value of the problem. Note that $V_{inj}$ is a constant dependent on the experimental conditions but it is not a parameter of the model.

With this expression, the limiting maximal tumor size of the Gompertz model is ${K=V}_{inj}e^{\alpha/\beta}$, that is independent of the choice of the initial value ${(t}_{I},V_{I})$.

In the population analysis we fixed ${(t}_{I},V_{I})=(0,V_{inj})$, while for the backward predictions we considered as initial data the (n-2)-th observation of the $i$-th animal ${(t}_{I},V_{I})=(t_{n^{i}-2}^{i},y_{n^{i}-2}^{i})$.

A revision of paragraph 2.2 has been done taking into account the comments mentioned above:

*“Note also that the value of K in the Gompertz model is independent of the initial data* $\left( t_{I},V_{I} \right)$*. The latter was considered to be* $\left( 0,V_{inj} \right)$ *when performing population analysis, while it was set equal to the observation* $y_{n^{i}-2}^{i}$ *of an animal i for backward prediction.”*

**2. Moreover, in both type of the Gompertz model (full and reduced) there arises a problem of dependance on V_inj, which is known in vitro experiments but uknown in vivo. This is probably the main problem considering the utility of the proposed method.**

As reported in the discussion, for clinical translation the injected volume $V_{inj}$ should be replaced by the volume of one cell $V_{c}={10}^{-6}$ cells. The reason why we opted for $V_{inj}$ instead of $V_{c}$ for analysis of our experimental data resides in the biological interpretation of the parameters $\alpha$ and $\beta$. Given the expression of the Gompertz model as in (1), $\alpha$ corresponds to the specific growth rate when the volume $V$ is equal to $V_{inj}$. This choice allowed us to compare the parameter $\alpha$ with the proliferation rate estimated from *in vitro* experiments (Section 3.2.2).

In clinical applications, $V_{inj}$ should be replaced by $V_{c}$ and we expect the parameter $\alpha$ to be close to the proliferation rate ($=\frac{\ln2}{T_{c}}$ with $T_{c}$ the length of the cell cycle).

**3. I have also found some editorial and language mistakes, like**

**- line 120 "number of injected" WHAT?**

**- line 127 "litterature" should be literature**

**- in Formula (6): what is p here? In line 168 p is the number of parameters!**

**- in line 176: "equation(7)" - lack of space.**

We thank the reviewer for these observations. These points have been addressed in the text.

**REVIEWER #3**

**The present manuscript reports a study of tumour growth using classical ODE models and in vivo data, in the mixed-effects modelling setup.**

**The authors compare three model types (exponential, logistic and Gompertz), perform population model fit and model selection, using standard goodness-of-fit criteria and visual comparison with data. Gompertzian model is found to provide best fit, and further it is suggested that one of the parameters (related to carrying capacity) can be fixed in the population, leaving only one parameter (related to the growth rate change) to have inter-individual variability, the model termed “reduced”.**

**The model is applied to evaluate the tumour initiation time from the last three measurement points of an animal: namely, these three points are used to evaluate individual parameters and the tumour size is back-traced to the known initial volume in order to evaluate the time. The best results are achieved using the reduced model with population priors and bayesian estimation of the individual parameters.**

**Eventually, the authors claim that this methodology can be used to evaluate “age of tumour”, i.e., time from its initiation in the clinical settings (while acknowledging that direct verification of this estimation is impossible).**

**This work is an interesting and well-designed exercise in data-driven modelling and model selection. The authors apply sophisticated methodology and derive various insights and observations. Yet, I have reservations on some of the suggested interpretations, and think that part of the conclusions and claimed prospects are too far-fetched.**

**1. It indeed can be interesting to evaluate the utility of different growth models for describing data in different contexts. However, I would not take the advantage of Gompertz model in this study to signify its universal prevalence, even for the in vivo data only. First, a proper comparison could include more flexible alternatives; e.g., both logistic and Gompertz are special cases of a generalized model (with an additional parameter). It is conceivable that such general model could provide better fit to data, in terms of AIC/BIC, notwithstanding additional degrees of freedom. Another possibility would be to evaluate the general model and to check whether the evaluated parameters indeed suggest that it is closer to the Gompertz formulation, rather than to pure logistic.**

We thank the reviewer for this valuable comment, which motivated us to conduct a population analysis of the generalized logistic model (provided in a supplementary file). As expressed in our answer to point 1) of reviewer 1), we obtained that the generalized logistic indeed provided good fits to the data but it lacked robustness of convergence in parameter estimation: starting from different initial parameter values the algorithm converged to different estimates. This is probably due to the larger number of parameters of the model leading to identifiability problems. In addition, for most of the fits the value of parameter $\nu$ (the power in the generalized logistic model) was found very small, with a corresponding value of $\alpha$ very large, indicating convergence to the Gompertz model.

We have added in the discussion the following comment on the generalized logistic model:

*“The logistic and Gompertz models belong to the same family of tumor growth equations and can be seen as specific cases of the generalized logistic model* $\frac{dV}{dt}=\alpha V\left( 1-\left( \frac{V}{K} \right)^{\nu} \right)$ *[40, 15]. We also analyzed the latter model, which demonstrated good descriptive power but lacked robustness of convergence (data not shown). Indeed, the SAEM algorithm converged to different estimates starting from different initial guesses of the parameters. This might be explained by the larger number of parameters (3) that led to identifiability problems. In addition, we found that values of ν able to describe the data were often very small (< 10−3), thus suggesting convergence to the Gompertz model.”*

**2. Second, The authors themselves suggest the possible properties of the data that make them more suitable for Gompertz curve – the way the exponential velocity decelerates makes the symmetric logistic curve less suitable. However, from the presented individual plots, it seems that the overall pattern is that the logistic model reaches early saturation, while Gompertz is almost not saturating at all. This suggests two questions: (1) did the authors verify that the parameter ranges for the logistic model allowed obtaining such late saturation (higher K and larger alpha)? (2) could it be that the model with decelerating growth but no saturation (e.g., V~t^alpha for alpha ~1-2) would provide also a good description of these data? Inspection of the pooled data charts suggests that the latter is a viable option.**

A detailed analysis to this comment is provided in the supplementary file.

For point (1) we tested multiple initial conditions for the parameters of the logistic curve, including large values of $K$ (supplementary file, section 3). Note that no constraint was imposed on parameters $K$ nor $\alpha$, thus allowing the algorithm to converge to the global maximum of the likelihood. Simulations were done starting from initial guesses of the parameters in the range of [1000, 3500] mm^3^ for $K$ and [0.1, 0.5] for$\alpha$. We found that the algorithm always converged to the same value of the parameters with small $K$ ($\simeq1300$ mm^3^, see Figure R4). This demonstrates that larger values of $K$ are unable to provide better fits to the data. Indeed, such values would also imply a slower slowdown of the specific growth rate $\frac{1}{V}\frac{dV}{dt}$ which would deteriorate the fit of the early data points, even with a larger $\alpha$.

For point (2), we provide a population analysis of the power law model (supplementary file, section 1). Although exhibiting good descriptive power, this model did not outperform the Gompertz or even reduced Gompertz in terms of AIC and BIC (Table R1).

The main focus of our manuscript is the application of the population approach and Bayesian estimation to the Gompertz model, in order to rigorously define the reduced Gompertz model. Thus we did not include the power law or generalized logistic in the manuscript as we believed that this would make it diverge from its main purpose.

**3. Further, it is known that there is a certain delay time between the xenograft implantation and initiation of its growth, just due to the time required for the cells to adjust to the new environment (and possible death of part of these cells). This is not taken into account here explicitly, and hence the Gompertz model gets its advantage from the possibility to accommodate this lag, which is not formally included between its assumptions.**

We thank the reviewer for this interesting observation. In the supplementary file we provide an analysis of the Gompertz model introducing a delay (section 4). The results did not differ significantly from the ones where no delay was considered, suggesting that the time needed for the cells to adapt to the new environment might be negligible in our case. On the other hand, we don’t have the proper data to address this question (see point 16).

**4. Considering the so-called “reduced Gompertz model” – I would not call it a reduced model, in order to not give a false impression that this is model with less parameters or different structure, while the whole change amounts to reducing the random component in the population model for one of the parameters.**

Our choice of calling it “reduced Gompertz model” is motivated by the fact that it has only one individual-level parameter, while the second one is fixed within a population. Therefore, once this population-level parameter $k$ has been determined (from a preliminary population study, or previous experiments), only one parameter remains to be fitted for a given individual growth curve. This can be seen in the following by a re-parameterization of the model:

$$\left\{ \begin{aligned} \frac{dV}{dt}= \left( \beta k-\beta\ln\left( \frac{V}{V_{inj}} \right) \right)V, \\ V\left( t_{I} \right)=V_{I}. \end{aligned} \right.$$

**Further, if one used a parameterization with beta and K, the equation becomes**

**dV/dt = beta*ln(K/V)*V,**

**from which it is clear that beta is the parameter that guides the growth rate (and not only alpha, as is claimed in the article).**

We do not claim that the growth rate depends only on $\alpha$ but rather that $\alpha$ is the *specific* growth rate (i.e. $\frac{1}{V}\frac{dV}{dt}$) *at the injection time*, while $\beta$ is a parameter that guides the exponential decay of the growth rate. Thus $\beta$ also impacts on the growth rate.

Considering the parameterization of the Gompertz model expressed by the reviewer $dV/dt=\beta\ln\left( K \right) V$, we would like to point that it also writes

$$\frac{dV}{dt}=\left( \beta\ln K \right)V-\left( \beta\ln V \right)V$$

from which it is apparent that for $V$ close to $1$, the quantity that drives the specific growth rate is $\beta\ln K$. If $V_{inj}=1$, then $K=e^{\frac{\alpha}{\beta}}$ and thus $\beta\ln K=\alpha$, consistently with our claim. For $V_{inj}\neq1$, noting that the expression above writes

$$\frac{dV}{dt}=\left( \beta\ln\left( \frac{K}{V_{inj}} \right) \right)V-\left( \beta\ln\left( \frac{V}{V_{inj}} \right) \right)V$$

shows similarly that the specific growth rate at $V=V_{inj}$ is $\beta\log\left( \frac{K}{V_{inj}} \right)=\alpha$.

**It is indeed an interesting observation that the parameter K (or equivalently, k) can be fixed in the population. However, given that this happens in the set of identical mice implanted with the same amount of identical tumour cells, in one experiment, this is hardly surprising. It would be far-fetched to conclude that K is a species / tumour type constant (and indeed, as noted by authors, it differs in the three analyzed datasets). Eventually, the translation of the ability to trace the history of tumor back from this highly uniform and controlled experimental setting (all in all, the individual tumours seem to grow very similarly and synchronously) into any clinical application is extremely uncertain. There is yet no well-settled theory on the typical individual history of human cancers, which may well differ from the case when the already significantly transformed cancer cell is implanted into an experimental animal. In simple words, the “growth law” followed by a clinical tumour can change significantly (both in parameters and structure) during disease progression, especially at early stages. Therefore, I suggest to moderate the optimistic prospects of using this approach in the clinical setup.**

We agree with the reviewer that clinical applications of our methodology have some limitations. We have added the following paragraph in the discussion to acknowledge the homogeneity of the data in controlled experimental settings and moderate the prospects in the clinical setting:

*“Our methodology might face multiple challenges for future clinical applications. First, it is indeed clearly difficult to fully characterize tumor kinetics in humans and only few studies support the evidence that it follows a gompertzian growth [8]. This is due to the limited number of available observations in the clinic and to the fact that saturation of human tumors is almost never reached, since it coincides with an advanced stage of the cancer where patients usually receive a treatment. Moreover, human tumor growth curves, even if limited to the same organ and histological type, might show a substantially larger variability than in in vivo experimental settings where cells from immortalized cancer cell lines are injected in genetically identical mice. Here, we have proven that a group of animals with similar features (such as tumor type) is characterized by a common tumor growth constant, that defines the saturation point. In the human setting, it could be interesting to analyze this constant as a function of some covariates (such as weight, sex, tumor type). Eventually, in the Gompertz model we haven’t considered that the initial phase of tumor growth might be affected by intrinsic stochasticity. Our choice was motivated by the large number of injected cells (of the order of 106) that allowed us to consider the initial variability to be negligible. For accurate clinical translation stochasticity should ideally be taken into account to model the initial stages of tumor growth.”*

**More technical remarks and suggestions:**

**5. p. 3 l. 82-85: please check formatting (superscript)**

Thank you for the remark. All the LM2-4LUC+ have been changed into LM2-4^LUC+^.

**6. p.3 l.109: alpha is not equal to the specific growth rate at t=t_I or t=0 in the logistic equation. In this paragraph it is also not clear what is the relation between t_{inj}, V_{inj} and t_I, V_I**

We thank the reviewer for the remark. In section 2.2 we have introduced a new parametrization of the logistic model, that is slightly different from the previous one:

$$\frac{dV}{dt}= \rho\left( 1-\frac{K}{V} \right)V,$$

where the relation between $\rho$ and the specific growth rate $\alpha$ at ${V=V}_{inj}$ is given by

$$\rho=\alpha\left( \frac{K}{K-V_{inj}} \right).$$

For small values of $V_{inj},$ $\rho$ tends to $\alpha.$

Moreover, we have clarified in text the relation between $t_{inj}$, $V_{inj}$and $t_{I}$, $V_{I}$ in section 2.2:

*We remark that the value of K in the Gompertz model is independent of the initial data* $\left( t_{I},V_{I} \right)$*. The latter was considered to be* $\left( 0,V_{inj} \right)$ *when performing population analysis, while it was set equal to the observation* $y_{n^{i}-2}^{i}$ *of an animal i for backward prediction.*

**7. p.4 l.130: the choice of the log-normal model for the parameters should be motivated**

In section 2.3 we added the motivation of the choice of a log-normal distribution for the parameters:

*“The choice of a log-normal distribution ensured the positivity of the parameters without adding any constraint. Moreover, the ratio of two log-normal distributions is a log-normal distribution.”*

**8. p. 4 l.134-137: for the reader who is not intimately familiar with the NLME field, there is not enough information here; please give some more details and add a reference to a good theoretical literature source**

We have added in text some details and references to the NLME algorithm:

*“This algorithm is a variation of the EM algorithm, where the expectation step is replaced by a stochastic approximation of the likelihood function [10]. This method has been proven to efficiently converge to the maximum likelihood estimator for nonlinear mixed effects models [11].”*

**9. p.4 l. 143-144: why do you need to shift the time scale?**

Indeed, it is not necessary to shift the time scale. We reformulated the definition of the age of the tumor in section 2.4.

**10. p. 5 l.154: “constant part σ1 was found negligible” there is no sufficient information to support this claim. Please explain how you evaluated this from Table 2. I have a suspicion (and indeed you mention this in the discussion) that the goodness-of-fit comparison is influenced by your choice of error model. Using proportional error, naturally emphasizes smaller values more, comparing to the additive model. Did you try to fit the data using only additive error?**

The facts that a constant error is inadequate to describe measurement error of tumor volumes, and that a combined error model should be preferred, has been previously proven in [15]. The proportional part of the error reflects the fact that the measurement error is larger for larger volumes. The constant additive part of the error plays a role in small measurements, since the measurement error of very small tumors should not be a function of the volume because of detectability limits.

We then found the constant part to be negligible for backward predictions. Indeed, in this case, only the last three measurements were considered and only the proportional part of the error played a significant role in the variability. For example, considering the Gompertz model, if the last three measurements of an individual were all >1000, the proportional part of the error was >120mm^3^ and the additive part of the error model was of the order of 20mm^3^, therefore considered negligible.

However, we agree with the fact that the last three measurements may not be large enough to consider σ_1_ negligible. We therefore performed again our analysis taking into account also the constant part of the error model and reported the results in Table 3 and Figures 4-5. Results did not show significant differences with respect to the previous ones, confirming that σ_1_ did not play a significant role in this case.

Nevertheless, for completeness purpose, we provide in the supplementary file to the reviewer the analysis of the breast volume data using an additive error model. While we agree with the reviewer that this could have had significant impact on the results, we still observed misspecification of the logistic model (Figure R7). The statistical distributions of the residuals of the Gompertz and reduced Gompertz models were also deteriorated, yet to a lesser extent. This was also confirmed by larger values of the AIC (7362 for the additive error model versus 7129 for the combined error model, see Table R6). We added one sentence in the discussion to acknowledge these additional elements.

**11. p. 5 l.163: please explain the formula for \hat{s}^{2,i}. is it the sufficient statistics for the error? Why the 3-p in the denominator?**

We have considered as statistic for the error of an individual *i*

$$\hat{s}^{2,i}= \frac{1}{3-p}{\sum_{j=n^{i}-2}^{n^{i}} \left( \frac{y_{j}^{i}-f\left( t_{j}^{i}; \theta^{i} \right)}{\sigma_{1}+\sigma_{2}f\left( t_{j}^{i}; \theta^{i} \right)} \right)}^{2}$$

where *3* is the number of observations per individual that we have considered to do backward predictions (now specified in the text).

In the expression of $\hat{s}^{2,i}$ in text (section 2.4.1) there was a missing sum that has been added.

**12. p. 5 l. 172: it is not clear whether V_I was sampled once per animal, or at each iteration of bayesian estimation of \theta_I**

For each animal, $V_{I}^{i}$

$V_{I}$ is sampled at each iteration of the Bayesian estimation, since it is considered as a random variable $V_{I}\sim N(y_{n^{i}-2}^{i}, \sigma_{1}+{\sigma_{2}y}_{n^{i}-2}^{i})$.

We have clarified this point in Section 2.4.2 that $V_{I}^{i}$ was sampled at each iteration of Bayesian estimation algorithm. In particular, the likelihood can be computed as follows:

$$P\left( \boldsymbol{y}^{i}|\boldsymbol{\theta}^{i} \right)= \int_{\mathbb{R}} P\left( \boldsymbol{y}^{i}|\boldsymbol{\theta}^{i}\boldsymbol{,}V_{I}^{i} \right)P\left( V_{I}^{i} \right)dV_{I}^{i}.$$

Since the sampling procedure depends on the evaluation of the likelihood, which relies on $V_{I}^{i}$, the initial volume $V_{I}^{i}$ was sampled from its distribution for each realization of the posterior distribution.

**13. p.8 legend to Figure 1: please add explanations to A and (especially B). how did you generate the distributions?**

The visual predictive checks were obtained by multiple simulations of the model. The time axis was then split into intervals and in each interval the empirical percentile of the observed data were compared with the respective predicted medians and intervals of the simulated data. The prediction distribution was obtained by multiple simulations of all individuals in the dataset, excluding the residual error.

We provide an explanation of the two plot types in caption of Figure 1.

**14. p.10 legend to Table 2: what is CV? is it estimation of \omega? Further, since all the parameters are estimated in log scale, it’s not clear, are these CV and RSE in the log scale or the real scale (and in the latter case they would be asymmetric)**

We have substituted the CVs with the standard deviations of the random effects $\omega$ (where $\log\left( \theta^{i} \right)\sim N(\log\left( \mu\right),\omega)$). For small values of $\omega$, the standard deviation of the logarithm of a parameter is equal to the coefficient of variation of the parameter (divided by 100). Indeed, considering $\eta^{i}\sim N(0,\omega)$ small, the following holds:

$$\theta^{i}=\mu exp(\eta^{i})\approx\mu(1+\eta^{i})$$

Therefore,

$$\frac{\mathrm{CV}\left( \theta^{i} \right)}{100}=\frac{std\left( \theta^{i} \right)}{\mu}\approx\omega.$$

The (relative) standard errors of the population parameters **µ** and **Ω** are obtained by a stochastic approximation of the Fisher Information Matrix. The RSE are in real scale but they are the relative standard errors of the estimates.

**15. p.10 l. 253: “The other data sets gave analogous results” Not clear, does it also mean, the same value of k? Or the fact that it can be fixed?**

The performances of the other two data sets were similar to the breast cancer data set in terms of goodness of fit and correlation between $\alpha$ and $\beta$, even if the constant limiting size was found different in the three data sets.

In the text we underline that the limiting sizes of the data sets are different.

**16. p. 10 Section 3.2.2 “Biological interpretation…”**

**I have several reservations for this discussion. First, if your suggestion is assumed, the V_{inj} at the initial stage (at t=t_{inj}) should in fact be set equal to \hat{V}_{inj} and the value of alpha should be re-estimated. At least, this is what you assumption entails.**

**On the other hand, leaving the parameters as they are, one could hypothesize that in the new conditions the cells reduce their division rate (which also sounds more plausible, from the biological point of view). Cf. my remark above about the lag in the growth after the implantation. I think this merits more elaborate analysis.**

We thank the reviewer for pointing these elements. We have substantially modified the section 3.2.2 in the intent to clarify our point. We agree with the reviewer that the way things were expressed was unclear, and that $\alpha$ should be re-estimated if we follow our assumptions, for instance. What is expressed now is that the parameter $\alpha$ should remain the value of the specific growth rate (SGR) at $V_{inj}$, even if this value $V_{inj}$ does not coincide with the actual number of cells that take (e.g., $V_{inj}=1$ and then $\alpha$ is the SGR when $V$ reaches $1$). Then, to give estimates of the latter quantity we propose a reformulation of the Gompertz model with the following hypotheses: 1) a variable (animal specific) initial condition (denoted $\hat{V}_{inj}^{i}$) representing the number of cells that take and thus satisfying $\hat{V}_{inj}^{i}<V_{inj}$ and 2) a fixed SGR at $V=\hat{V}_{inj}^{i}$ given by $\lambda$ (the *in vitro* proliferation rate).

We also agree with the reviewer that a lag time could represent an alternative explanation. We have added the following comment in section 3.2.2:

*“Alternatively, these results might also be explained by a time lag between the cell implantation and the initiation of tumor growth, due to the time needed by the cells to adapt to the new environment [40]. However, the two interpretations are indistinguishable in our case and might require a more elaborate analysis with specific data.”*

**17. p. 12 Figure 3 legend: I suspect the panel enumeration got mixed. The same is true for figures S5, S6**

Thank you for the remark. Indeed, captions of Figure 3, S5 and S6 have been modified.

**18. p. 14 Table 3 legend: please explain the PI column**

In caption of Table 3 we explain the PI column (i.e. prediction interval that we have considered as a measure of precision).

**Recommendation: I think the work is good and merits publication, after the above suggestions and concerns are addressed**

We thank the reviewer for his/her positive appreciation of our work.

Sincerely,

**Dr Sebastien Benzekry, Corresponding Author.**
