# Supplementary material for: Population modeling of tumor growth curves and the reduced Gompertz model improve prediction of the age of experimental tumors
Source: PLoS Comput Biol. 2020 Feb 25;16(2):e1007178. doi: 10.1371/journal.pcbi.1007178 (PMC7059968; doi:10.1371/journal.pcbi.1007178)
Supplement: Attachment — Submitted filename: minor_revision.docx [file pcbi.1007178.s017.docx]

**Zvia Agur, PhD
Guest Editor
PLOS Computational Biology

Feilim Mac Gabhann
Editor-in-Chief
PLOS Computational Biology**

Dear Pr Agur,

Thank you for your additional comments. We have made every effort to address them. In addition to modifications related to your comments below, we have: (i) deposited the data to devoted, separate and publicly available repositories and (ii) added statistical significance tests for comparison of the error distributions in Figure 5 (and related supplementary Figures). We hope that our manuscript can now be considered for publication in PLOS Computational Biology.

**1. One reviewer still has reservations on the implications of the assumption that k may be constant. The reviewer trusts your findings that from the statistical point of view, this can be assumed here without loss of goodness of fit. However, he doubts your claim that this is a universal property. Simply put, in this model we have carrying capacity K = Vinj exp(k). If k is fixed for the tumor type and animal, this means that by repeating this experiment, and injecting say 0.1*Vinj one will obtain a 10 times smaller carrying capacity; or inversely, injecting 10 times more cells will allow tumor to grow asymptotically to a 10 times larger size. This dependence of saturation level on the initial size seems biologically unsound.
The reviewer does not think this invalidates your results, but that it calls for more careful interpretation. Possibly, the real "natural constant" is K, and the value of k would change if you inject larger number of the same cells into the same animals. This concern should be elaborated in the discussion, especially when you envisage the possible use of the model to trace the tumor growth back in time to the size of 1 cell.**

We agree with the interesting observation of the reviewer. We have clarified in the discussion that the parameter k depends on the species, tumor type and number of injected cells:

*Interestingly, we found different values of the carrying capacity K for the breast and the lung cancer cell lines measured by volume (K = 2600 mm3 and 12300 mm3, respectively), in contrast with previous claims [11]. This suggests that there might not be a characteristic saturation point within a species [48] but the carrying capacity could be a typical feature of a tumor type in an animal model. From (10), the population constant k depends on the value of the parameter Vinj, therefore it cannot be viewed as a universal constant of tumor growth. However, it can be considered as a common trait within a species with similar characteristics (such as tumor type and value of Vinj). We used the formulations of the Gompertz (3) and reduced Gompertz (10) in order to define α as the specific growth rate at injection, which could be compared to the in vitro proliferation rate λ. This could be leveraged clinically to predict past or future tumor growth kinetics based on proliferation assays, derived from a patient’s tumor sample.*

**2. The second concern the reviewer raises is the insufficient review of previous works on “mathematical models for tumor growth, which have been previously studied and compared at the level of individual kinetics and for prediction of future tumor growth” (line 53). Please note that there is previous work on the subject, e.g., Kronik N., Kogan Y., Elishmereni M., Halevi-Tobias K., Vuk Pavlović S., Agur Z. Predicting Effect of Prostate Cancer Immunotherapy by Personalized Mathematical Models PLoS One 2010 5(12) (and it deals with the clinical data) and several other publications in the last 10 years. Please, study the literature and include previous work on the subject appropriately in your review.**

In the introduction we have expanded our literature review. In particular, thank you for reminding us about the excellent paper by Kronik et al. which we actually we aware of but had forgotten to mention in our introduction. Although dealing with PSA kinetics and immunotherapy, it indeed deserves to be cited for the investigation conducted on the predictive power.

*A calibrated model of lymphoma tumor growth has also been introduced and used for predictions in [26]. More complex mechanistic models have been proposed to investigate the link between biological processes and tumor growth dynamics and perform predictions, including angiogenesis [27] and solid stress [28]. A model for tumor-immune interactions has been developed and validated in [29, 30], demonstrating its ability to predict future prostate specific antigen dynamics based on several pre- and post-treatment initiation data points. Mathematical models of tumor growth inhibition were presented to assess tumor size dynamics in colorectal cancer [31] and adult diffuse low-grade gliomas [32]. Spatial models have also been widely proposed in a theoretical context but few of them have been compared to data (see [33] for an example on thyroidal lung nodules and [34, 35] for gliomas).*

Sincerely,

**Dr Sebastien Benzekry, Corresponding Author.**
